# Computed Tomographic and Ultrasonographic Features in Three Dogs with Infected Uterus Masculinus and Concurrent Genital Neoplasia

**DOI:** 10.3390/ani15223357

**Published:** 2025-11-20

**Authors:** Clara Pagá-Casanova, Laura Librán-Ferreira, Vicente Cervera-Castellanos

**Affiliations:** Hospital Veterinario AniCura Valencia Sur, Avenida Picassent 28, 46460 Silla, Spain

**Keywords:** canine, disorder of sexual development, male pyometra, persistent Müllerian duct syndrome

## Abstract

Uterus masculinus is a rare disorder of sexual development in males, characterized by the presence of tubular female genitalia. Diagnostic imaging reports of infected uterus masculinus are limited. We describe the ultrasonographic and computed tomographic findings in three dogs, all presenting with abdominal distension, pain, and systemic infection. Imaging consistently revealed a fluid-filled, bicornuate structure arising from the prostate. In two dogs, the horns extended through the inguinal rings to the scrotal testes; in the third, with a prior left orchiectomy, both horns were intra-abdominal, the right ending in a peritoneal mass. Surgery and histopathology confirmed infected uterus masculinus, with *Escherichia coli* isolated from urine in all dogs and from the structure in two. Two dogs had Leydig cell tumors, one with concurrent uterine neoplasia; the third had an ovarian or ovotesticular granulosa cell tumor. Although rare, infected uterus masculinus is potentially life-threatening and should be considered in male dogs with abdominal pain, distension, or systemic infection. This is the first case series describing combined ultrasonographic and tomographic features of infected uterus masculinus, including novel findings such as cervix-like mural narrowing and fluid–fluid levels. It is also the first imaging description of an ovarian or ovotesticular tumor in a dog with uterus masculinus.

## Simple Summary

Uterus masculinus is a rare disorder in which male dogs develop female reproductive organs. We describe three cases of infected uterus masculinus, all presenting with abdominal swelling, pain, and signs of infection. Abdominal ultrasonography and computed tomography revealed a fluid-filled, bicornuate abdominal structure connected to the prostate in all cases. In two dogs, each horn extended into the scrotum next to the testes, while in the third, both horns were entirely inside the abdomen, one of them attached to a large mass. Surgery and laboratory analyses confirmed an infected uterus masculinus, with the bacterium *Escherichia coli* detected in the urine of all dogs, along with diverse genital tumors. Although rare, this condition should be considered in male dogs showing abdominal swelling, pain, or infection. The imaging descriptions provided here may help veterinarians recognize this uncommon, so far poorly described disorder and treat affected dogs more effectively.

## 1. Introduction

Disorders of sexual development (DSD) arise from abnormalities occurring at any of three developmental stages: chromosomal, gonadal, or phenotypic [1,2,3,4]. During embryogenesis, the paired Müllerian (paramesonephric) ducts normally form the genital tubular system (oviducts, uterus, and cranial vagina), while in males, they regress. Failure of Müllerian duct regression leads to the persistence of female tubular genitalia, a condition known as uterus masculinus (UM) [2,3,4,5,6]. In dogs, UM is typically associated with persistent Müllerian duct syndrome (PMDS), formerly classified as male pseudohermaphroditism, which has a familial predisposition in Miniature Schnauzers [5,6,7,8].

Terminology remains controversial both in human and veterinary literature. UM has often been used interchangeably with the terms “prostatic utricle” and “paraprostatic cyst” [9,10,11,12,13,14,15,16]. The prostatic utricle is a small, blind opening at the level of the colliculus seminalis with no specific function [14,17], whereas paraprostatic cysts are fluid-filled structures extending from the prostate into the peritoneum [10]. Because the UM has been documented to communicate with the urethra, the term “cyst” is considered misleading and should be avoided [11]. Previous studies recommend using the term “uterus masculinus” [18], which we adopt throughout this report.

The presence of UM is often clinically silent but may become evident due to associated complications such as cryptorchidism, infertility, neoplasia, UM distension, or genitourinary infection [2,3,4,9]. Although there are several descriptions of UM and infected UM (IUM), reports including the abdominal ultrasonographic (AUS) and computed tomographic (CT) features of confirmed IUM remain scarce in dogs [18,19,20]. Malignancies associated with UM have also been described, most commonly testicular Sertoli cell tumors [7,8,9,10,18,20,21,22,23,24,25,26,27,28,29].

Given these limitations, greater awareness and detailed characterization are needed to enhance diagnostic accuracy and clinical management of IUM. This report aimed to describe the AUS and CT findings of IUM in three adult male dogs. To the authors’ knowledge, this is the first case series to document the combined AUS and CT features of IUM in dogs, in association with bacteriuria and diverse genital neoplasia, including an ovarian or ovotesticular tumor.

## 2. Materials and Methods

In this retrospective study, cases of IUM were identified from AUS and CT reports obtained at AniCura Valencia Sur Veterinary Hospital between June 2019 and June 2025. CT studies performed at external centers but subsequently reviewed and reported at the same institution were also included. A total of 3216 AUS and 302 CT reports involving complete abdominal examinations from 2733 male dogs were reviewed.

The database was searched for male dogs using the keywords “Müllerian duct”, “paramesonephric duct”, “uterus”, “pyometra”, and “pseudohermaphroditism”. Four suspected cases were identified based on clinical findings and imaging results. One case was excluded due to the absence of surgical or histopathological confirmation, leaving three cases with confirmed IUM diagnoses for inclusion.

Data retrieved from medical records comprised signalment, clinical history, physical examination, blood analyses, urinalysis, urine bacterial culture, AUS images and reports, CT images and reports, surgical reports, histopathology images and reports, and UM bacterial culture results. Different clinicians performed clinical history collection, physical examination, and sample collection. All data were available for the three dogs, except for AUS images and UM culture results in case 3, and photomicrographs were only available for case 3. No intraoperative or macroscopic photographs were available. Detailed histopathology reports, conducted by different European Board of Veterinary Specialisation (EBVS^®^) certified pathology specialists, were available for all cases. Diagnoses were rendered according to routine small-animal surgical pathology criteria.

Each included AUS examination was performed by a diagnostic imaging resident and a board-certified veterinary radiologist. The patients were conscious during the AUS examinations, which were conducted in both left and right lateral, as well as dorsal recumbencies. All AUS studies were performed using a Canon Aplio i600 unit (Canon Medical Systems, Tustin, CA, USA), equipped with convex (9–10.8 MHz) and linear (12–18 MHz) transducers.

CT examinations were performed under general anesthesia, with the dogs positioned in ventral recumbency, using 16-slice multidetector scanners. Cases 1 and 2 were scanned using a GE Medical Systems Revolution ACT unit (GE Medical Systems, Chalfont St. Giles, Buckinghamshire, UK), while case 3 was scanned on a Canon Medical Systems Aquilion Start unit (Canon Medical Systems, Tustin, CA, USA). CT studies were acquired with a slice thickness of 1.25 mm, an interval of 0.625 mm, pitch values of 0.562–0.928, a tube rotation time of 0.75–1 s, a tube voltage of 100 kVp, and a tube current of 50–80 mA. All protocols included standard soft tissue and bone reconstruction algorithms of the entire abdomen, before and after intravenous administration of iodinated contrast medium, with unspecified phase protocols. Differences in technique between cases 1 and 2 compared to case 3 were due to the latter study being performed at an external center before referral. Because both the referring center and our institution lack a contrast injector, contrast timing is not standardized and typically not recorded unless clinically required. The image quality of the three studies was considered adequate for interpretation and pre-surgical diagnosis.

AUS and CT images were reviewed by one board-certified veterinary radiologist and two diagnostic imaging residents. All assessments were reached by consensus among reviewers, who were aware of the final diagnosis at the time of interpretation. Image review was conducted using a dedicated workstation (Horos, version 3.3.6, Horos Project, Annapolis, MD, USA). CT interpretation was based on the available pre- and post-contrast soft tissue algorithm reconstructions. A standard soft tissue window (window width 400; window level 40) was employed, with manual adjustments applied as needed to optimize visualization of high or low-contrast structures. The following characteristics were evaluated: UM shape, course, and extent; wall thickness, contour, echogenicity, attenuation, and contrast enhancement; luminal contents attenuation, echogenicity, and degree of homogeneity; presence, location, size, and shape of gonads and gonadal masses; prostatic size and texture; urinary bladder wall and luminal characteristics; presence and echogenicity of free abdominal fluid; and regional lymphadenomegaly.

## 3. Results

Three adult male dogs with acute nonspecific complaints were included. Blood samples were collected via jugular venipuncture, and a complete blood count, biochemistry panel, electrolytes, and coagulation profile were performed. These data are summarized in Table 1.

### 3.1. Abdominal Ultrasonographic Findings

**Figure 1:**
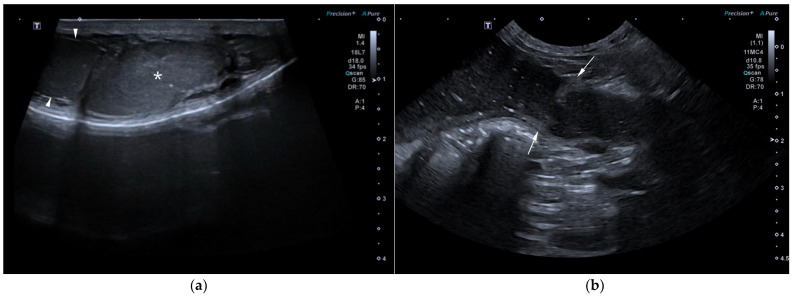
Abdominal ultrasound (AUS) of case 1: (**a**) Oblique view of a uterine horn (arrowheads) within the scrotum, with the testis in the center (asterisk) and the epididymis on the right side of the image; (**b**) Longitudinal view of the caudal aspect of the uterus masculinus (UM) showing a focal cervix-like mural narrowing ventral to the descending colon (arrows).

**Figure 2:**
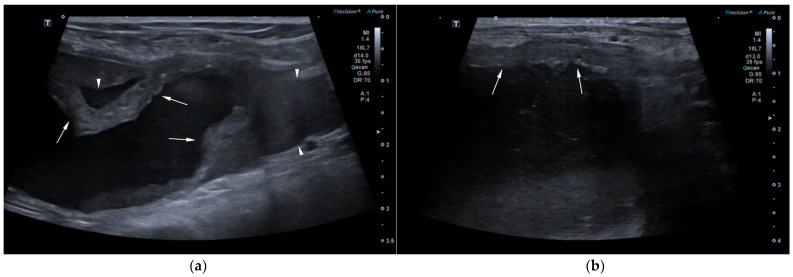
AUS of case 2: (**a**) Longitudinal view of the caudal UM, showing irregular wall margins (arrows), intraluminal inhomogeneous fluid, and free echogenic peritoneal fluid (arrowheads); (**b**) Detailed view of the UM wall demonstrating luminal irregular margins (arrows).

**Figure 3:**
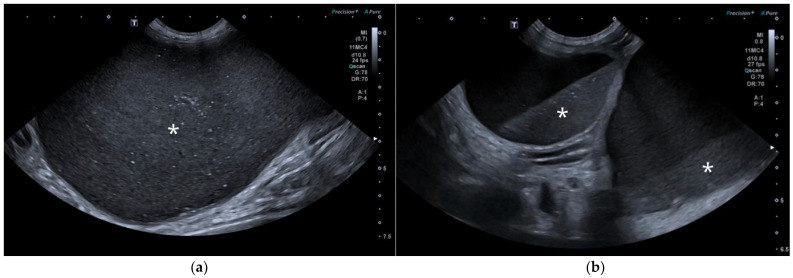
(**a**) AUS of case 1; transverse view of the most dilated UM segment containing inhomogeneous echogenic fluid; (**b**) AUS of case 2; oblique view showing two sections of the UM containing a moderate volume of sedimented material (asterisks), creating marked fluid–fluid levels.

AUS in all three dogs revealed a fluid-filled cavitary structure originating in the caudal peritoneum between the colon and urinary bladder and extending cranially through the mid-abdomen, causing a mass effect. In cases 1 and 2, the lesion continued bilaterally as paired, thin tubular extensions coursing caudally through the inguinal rings adjacent to each testis (Figure 1a). In case 1, a focal mural narrowing resembling a uterine cervix was noted caudally (Figure 1b). The wall was thin and smooth in case 1, while in case 2 it was irregular, especially in its caudal aspect (Figure 2a,b). No information was available regarding the course of the UM horns or wall appearance for case 3. The luminal content consisted of echogenic, inhomogeneous fluid in all cases (Figure 3a); in case 2, sedimentation created fluid–fluid levels (Figure 3b).

In cases 1 and 2, both testes were scrotal, with multiple bilateral nodules in case 1 and a single nodule in the left testis in case 2. Case 3, previously orchiectomized on the left side, presented with a large, heterogeneous right-sided cranial abdominal mass.

Additional findings in all dogs included: enlarged, heterogeneous, cystic prostate; echogenic sediment in the urinary bladder; free peritoneal fluid [moderate and echogenic in case 2 (Figure 2a); mild and anechoic in cases 1 and 3]; and bilateral medial iliac lymphadenomegaly (moderate in cases 2 and 3; mild in case 1).

### 3.2. Abdominal Computed Tomographic Findings

In all cases, the full extent of the abnormal structures could not be assessed sonographically due to their large size; therefore, CT was performed to allow a more complete evaluation and assist in surgical planning in cases 1 and 2. In case 3, CT had been performed earlier the same day at the referring center.

**Figure 4:**
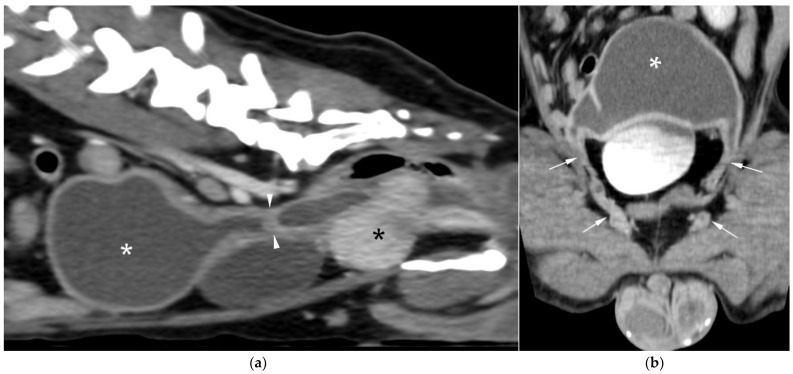
Computed tomography (CT) of case 1: (**a**) Slightly oblique longitudinal view showing the UM, extending from the prostate gland (black asterisk), passing dorsally to the urinary bladder, at which level a cervix-like mural narrowing (arrowheads) is identified, with a more distended portion of the UM (white asterisk) cranial to it; (**b**) Slightly oblique dorsal view showing the distended UM (asterisk) cranial to the contrast-filled urinary bladder, with the uterine horns coursing caudally on both sides and running through the inguinal rings (arrows).

**Figure 5:**
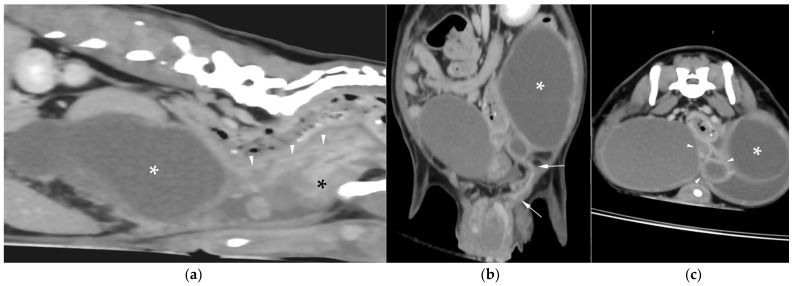
CT of case 2: (**a**) Slightly oblique longitudinal view showing the UM, extending from the prostate gland (black asterisk), not distended at this level (white arrowheads), through the mid-caudal abdomen, where a more distended portion of the UM (white asterisk) is identified. Ventral to it, there is a scant volume of peritoneal free fluid; (**b**) Dorsal view showing the UM (asterisk) on the left, with caudal extension of the left uterine horn through the inguinal ring into the scrotum (arrows), and the urinary bladder positioned on the right; (**c**) Transverse view depicting the urinary bladder on the right and three transverse sections of the UM (asterisk) on its left, surrounded by free peritoneal fluid. The two UM sections closest to the prostate show mild distention and irregular wall thickening (arrowheads).

**Figure 6:**
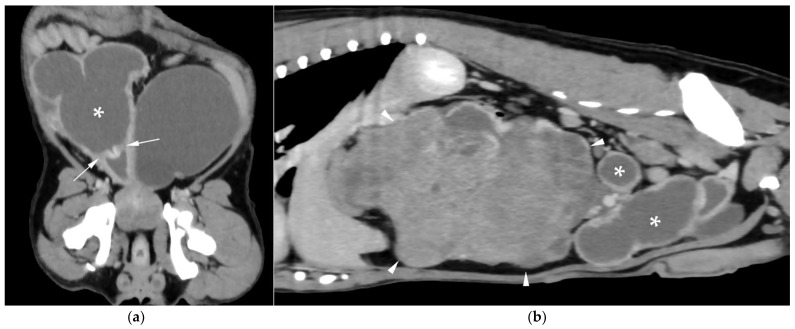
CT of case 3 (**a**) Dorsal oblique view showing the UM (asterisk) on the right, with a cervix-like mural narrowing (arrows) and cranially extending paired uterine horns. The urinary bladder is positioned to the left, and the prostate lies caudal to them; (**b**) Parasagittal view demonstrating a large right-sided cranial peritoneal mass (arrowheads) with multiple sections of the UM (asterisks) caudal to the mass.

CT revealed a large cavitary structure arising from the craniodorsal aspect of the prostate and extending cranially through the mid-abdomen in each dog (Figure 4a, Figure 5a and Figure 6a). In cases 1 and 2, the structure consisted of a large central cavity, with bilateral horns, coursing caudally through the inguinal rings to each scrotal testis (Figure 4b and Figure 5b). The horns were fluid distended, except for the right horn in case 2, in which no lumen could be identified. In case 3, a Y-shaped bicornuate structure was identified (Figure 6a); the right horn terminated in a cranial peritoneal mass, while the left one ended in the mid-caudal abdomen.

All lesions had thin walls with moderate contrast enhancement, slightly irregularly marginated in cases 2 (Figure 5c) and 3, and smooth in case 1. In cases 1 and 3, moderate dilation involved the entire organ, with a focal mural narrowing resembling a cervix observed at the caudal aspect of the structure (Figure 4a and Figure 6a). In case 2, only minimal dilation of the most caudal portion of the UM was noted (Figure 5a). The luminal content was mildly hyperattenuating (10–20 Hounsfield units) in all cases. It was homogeneous except in the cranial portion of the UM of case 2, where attenuation was slightly higher in the gravity-dependent ventral aspect (20 Hounsfield units) compared with its dorsal aspect (11 Hounsfield units). In case 1, small dorsal bubbles of gas were also noted, likely due to prior drainage at the referring center.

In case 1, both testes were heterogeneous with irregular, ill-defined multifocal lesions after contrast; in case 2, a nodule was present in the left testis. In case 3, in continuation with the cranial end of the right uterine horn and intimately associated with engorged right gonadal vessels, a large, irregular, heterogeneous mass with moderate contrast enhancement was identified, causing a regional mass effect (Figure 6b). No structures compatible with a left gonad were detected in case 3, consistent with the previous unilateral orchiectomy.

Additional findings in all cases included: an enlarged, heterogeneous prostate with small hypoattenuating foci after contrast administration; free peritoneal fluid (moderate in case 2, mild in cases 1 and 3); and iliosacral lymphadenomegaly (moderate in cases 2 and 3, mild in case 1). Enlarged mammary glands and nipples were also noted in case 3.

### 3.3. Ancillary Diagnostics, Surgical Management, and Histopathological Findings

Urine samples were collected by ultrasound-guided cystocentesis in each dog at the time of the AUS examination. Urinalysis and bacterial culture revealed active sediment with *Escherichia coli* isolated in each case. Ultrasound-guided fluid aspiration was obtained from the scrotal portion of a uterine horn in case 1 and from the free peritoneal fluid in case 2. Both samples confirmed septic exudate with bacilli.

All three dogs underwent surgical excision of the UM. In case 3, removal of the peritoneal mass and fine-needle aspiration of the medial iliac lymph nodes were also performed. Cases 1 and 2 additionally underwent prescrotal orchiectomy. No intraoperative evidence of UM perforation was found in any case. Culture of the UM fluid was performed in cases 1 and 2, yielding *E. coli*. The three dogs had an uneventful recovery.

On histopathology, case 1 revealed tubular structures containing eosinophilic proteinaceous material, leukocytes, erythrocytes, and karyorrhectic debris, lined by extensively ulcerated simple cuboidal to columnar epithelium. The submucosa and smooth muscular layers contained abundant inflammatory cells, and the muscle layer also harbored tubular structures containing spermatocytes. Findings were consistent with persistent Müllerian duct remnants with ulcerative, suppurative metritis.

In case 2, a cavitary structure containing abundant cellular debris and fibrin was observed. It was lined by ill-defined polygonal to globoid cells forming packs and papillae, with frequent binucleation, few atypical mitoses, and occasional early squamous differentiation without keratin. Cylindrical epithelium lacking cilia lined one edge of the sample. It was bounded by fibrovascular stroma and a thin smooth muscle layer. Tubular structures extended into the stroma, lined by simple cuboidal epithelium, associated with fibrous desmoplastic reaction and extensive granulation tissue infiltrated by neutrophils, macrophages, hemorrhage, fibrin, and edema. These features were consistent with an infiltrative malignant neoplasm of persistent Müllerian duct remnants with suppurative, fibrinous inflammation.

In cases 1 and 2, the testes showed reduced spermatogenesis, with fibrosis and mineralization in case 1. Well-demarcated masses composed of polygonal cells arranged in nests and cords were observed in both testes in case 1 and in the left testis in case 2. These findings were consistent with diffuse testicular degeneration and Leydig cell tumors. No evidence of endometrial hyperplasia or ovarian tissue was detected in cases 1 and 2.

**Figure 7:**
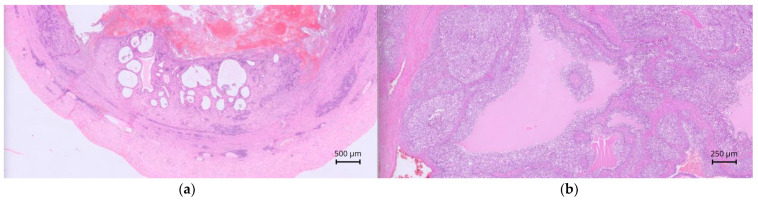
Histopathological images of case 3, hematoxylin-eosin staining. (**a**) 2× magnification. Tubular organ showing an outer circumferential muscular layer (myometrium) and an inner mucosal layer with dilated glands (endometrium). Cellular debris and hemorrhage are present within the lumen; (**b**) 5× magnification. Granulosa cell tumor composed of neoplastic cells forming irregular cystic cavities with central follicular fluid and peripheral clusters of neoplastic cells, resembling tertiary ovarian follicles.

In case 3, the lesion contained an intense neutrophilic, hemorrhagic, and macrophagic luminal infiltrate. The mucosa was extensively ulcerated and necrotic, with distended glands, and a peripheral muscular layer was noted. The wall contained plasma cells, lymphocytes, and neutrophils (Figure 7a). The peritoneal mass consisted of an organized multilobular proliferation divided by broad fibrotic bands, with large peripheral vessels and clusters of vacuolated cuboidal cells. Marked anisocytosis, anisokaryosis, and a high mitotic index were noted (Figure 7b). These features confirmed persistent Müllerian duct remnants with cystic endometrial hyperplasia and ulcerative, suppurative metritis. The mass was consistent with an ovarian granulosa cell tumor. Although no testicular tissue was noted, an ovotesticular origin could not be fully excluded. Cytology of the medial iliac lymph nodes in case 3 revealed abundant round or fragmented nucleated cells, eosinophilic background material, and fine cytoplasmic vacuolization, confirming metastatic lymphadenopathy from the granulosa cell tumor.

## 4. Discussion

This study describes the clinical, diagnostic imaging, and histopathological findings in three male dogs with confirmed IUM. Two had Leydig cell tumors, one with an additional diffuse uterine tumor, and the third had an ovarian or ovotesticular granulosa cell tumor.

Comprehensive DSD characterization requires genetic, histologic, and hormonal testing [1,2,3,4]. In the present study, genetic or hormonal testing were not performed, and histopathologic analysis of the scrotal gonad in case 3 was unavailable. Therefore, definitive DSD classification of these cases could not be established.

The pathogenesis of IUM in dogs remains incompletely understood, with both infectious and endocrine-mediated mechanisms likely being involved. A patent communication between the UM and the prostatic urethra has been reported in several cases, potentially allowing UM dilation, ascending infection, and the UM to act as a reservoir for pathogens [3,4,5,6,9,19]. Urinary tract infections (UTI) in dogs most commonly develop due to pathogens ascending from the urethra, with *E. coli,* a commensal bacterium of the external genitalia, being the most frequent causative agent [30]. IUM with concurrent UTI has been previously documented [5,19,24]. In our study, all three cases had *E. coli* bacteriuria, and in two dogs, *E. coli* was also isolated from the UM, supporting a presumed ascending infection pathway. Confirmation of this communication, which could have been assessed using contrast diagnostic imaging techniques or intraoperatively, was not available.

Prior reports also describe endocrine-responsive changes in the UM, which may precede pyometra. Endometrial hyperplasia of the UM has been associated with hyperestrogenism, typically secondary to Sertoli cell tumors, but also possible with other testicular tumors or unrelated conditions [9,10,11,21,22,27]. Hyperprogesteronemia has been reported in some cases of canine UM, one of which was infected and another with intraluminal content of unknown nature [11,18]. In female dogs, these hormonal changes have been linked to endometrial hyperplasia, with increased glandular tissue promoting mucoid accumulation within the UM lumen, a predisposing factor for pyometra [11,18,19]. In the present study, cystic endometrial hyperplasia was noted only in case 3, possibly due to hormonal influence from the granulosa cell tumor or preexisting ovarian tissue [31]. Cases 1 and 2 lacked histopathological evidence of endometrial hyperplasia or overt signs of feminization. Hormonal status was not assessed in any case, and further etiologic interpretation remains beyond the scope of this study.

The anatomic features of UM have been studied through direct observation, contrast radiology, AUS, and CT. The UM is reported as either a single pouch or, more commonly, a bicornuate tubular structure, resembling a true female uterus, extending cranially from the craniodorsal aspect of the prostate. When present, the horns can be fully intra-abdominal or extend through the inguinal rings in dogs with scrotal testes [9,10,13,16,18,19,20,21,22,23,24,25,26,27,28,32,33]. On AUS, the UM wall is isoechoic to the urinary bladder wall, with smooth to slightly irregular luminal margins, and mild fluid distension can be detected incidentally [11].

Because uncomplicated UM is typically silent, it is often diagnosed in older dogs prompted by UM distension and/or genitourinary inflammatory disorders [2,3,5,6,9,27] as in our series. UM distension can occur secondary to hydrometra [22,28], urometra [13,16], mucometra [21,25], or pyometra [6,18,19,24,27,28]. Reported clinical complaints in these cases include abdominal distension and pain, urinary and gastrointestinal signs, and evidence of infection [2,9,24,25,26]. All of our cases presented with abdominal distension, pain, gastrointestinal signs, and systemic infection; only one had urinary signs attributable to UTI. Prostatomegaly, which is a common finding on exploration [9,11,27], was present in all three dogs, along with external abnormalities (testicular tumors in two dogs and gynecomastia in the third).

Diagnostic imaging descriptions of significant UM distension include the AUS findings of hydrometra [22], and combined AUS and CT findings of urometra [13,16] and mucometra [21]. The imaging-based diagnosis of IUM using techniques different from radiography is rarely documented in dogs. Four reported cases based solely on AUS are available, although characterization of the intraluminal fluid and bacterial culture were performed in only one case [18,20]. Crosby et al. [19] provided the most comprehensive imaging description, employing both AUS and CT, although the latter was limited to CT urogram and retrograde contrast studies after a prior surgery with partial excision of the UM. The CT findings of IUM have also been recently described in a cat [34].

Consistent with the most common anatomical conformation, all three dogs exhibited a bicornuate UM. The course of the horns depended on the gonadal position, looping caudally through the inguinal rings in dogs with scrotal testes, and remaining fully intra-abdominal in case 3; in this dog, the location of the left uterine horn before orchiectomy was unknown. Two cases exhibited a cervix-like mural narrowing in the caudal portion of the UM. To our knowledge, this is the first diagnostic imaging documentation of a cervix-like UM mural narrowing in dogs, previously described only on gross anatomy [5,6]. Identification of this feature, or the presence of uterine horns, while not specific to IUM, may help distinguish UM-origin lesions from other cavitary structures.

Echogenic content within the UM has been previously described in two cases of IUM in dogs and one cat [18,19,34], as well as in a case of urometra in a male dog [13], the latter also containing loosely structured material. However, marked sedimentation of the UM contents, creating fluid–fluid levels, has not been previously reported, and may, when considered alongside a compatible clinical history, be supportive of IUM.

Malignancy of the persistent Müllerian remnants has been described in only two dogs: an adenocarcinoma of the UM involving the prostate [33] and a multicentric lymphoma affecting the UM [11]. Neither case showed specific features of neoplasia; however, the adenocarcinoma was associated with irregular, wavy walls with peripheral contrast enhancement [33], and the lymphoma exhibited irregular, hypoechoic walls [11], both cases with luminal distension. A leiomyoma originating from the UM has also been described [8]. In human medicine, although rare, PMDS carries an increased risk of tumors arising from Müllerian remnants [35,36]. The diffuse malignant uterine tumor diagnosed in case 2 had not been suspected based on AUS or CT, with endometrial hyperplasia or endometritis initially prioritized. However, the irregular walls of the UM were similar to the two aforementioned cases of UM malignancy.

PMDS in dogs is associated with cryptorchidism in approximately 50% of cases [1,3,4]. In humans, Müllerian remnants may mechanically impede testicular descent, whereas the cause of cryptorchidism in dogs with PMDS remains unclear [29]. Cryptorchidism greatly increases the risk of testicular neoplasia, with Sertoli cell tumors being the most common in cryptorchid and PMDS dogs, followed by seminomas [7,8,9,10,18,20,21,22,23,24,25,26,27,28,29]. Leydig (interstitial) cell tumor rates are similar in cryptorchid and scrotal testes [29]. Hyperestrogenism is a common paraneoplastic syndrome associated with canine testicular tumors, more prevalent with Sertoli cell tumors, especially if intra-abdominal, but rarely associated with seminomas or Leydig cell tumors [31]. Two dogs in this report had Leydig cell tumors with no signs of feminization. Case 3 was feminized, possibly by the granulosa cell tumor or ovarian tissue [31]. A comparable report describes the histopathological findings of a male dog with an ovarian adenocarcinoma, testicular atrophy, and pyometra [37]. No other references to concurrent UM and ovarian or ovotesticular tumors could be retrieved.

This case series provides the first combined AUS and CT descriptions of *E. coli*–associated IUM in multiple dogs, with concurrent bacteriuria. Novel diagnostic imaging features included the identification of a cervix-like mural narrowing and intraluminal fluid–fluid levels. It also represents the first diagnostic imaging description of an ovarian or ovotesticular tumor associated with UM. These findings expand the limited existing literature and provide diagnostic clues to recognize and characterize this rare process. Imaging findings were strongly supported by surgery, bacterial culture, and histopathology.

The main limitations of this study stem from the small sample size and its retrospective design. As the study was conducted in a referral center, selection bias is likely, and true prevalence cannot be estimated; some cases may also have gone undetected. The limited number of cases precludes generalization of the findings, as some might be overrepresented or others might remain undetected. Larger studies are needed to better define imaging features, prevalence, and associated malignancies.

The retrospective nature of the study prevented review of AUS images and UM culture in one case and limited the inclusion of histopathologic images in two cases. Histopathologic diagnoses were made by different pathologists without centralized review, which may have introduced interpretative variability. Although CT protocols differed slightly, quality was considered adequate in all cases. The reviewers were not blinded to the final diagnosis at the time of assessment. Although interpretation bias is possible, the same reviewers who performed the initial AUS and CT examinations had already identified IUM as the most likely diagnosis before surgical or histopathologic confirmation, minimizing retrospective bias.

Genetic and hormonal testing, required for complete DSD characterization [1,2,3,4], were unavailable. Finally, patent communication between the UM and the urethra was not confirmed, and UM contents were not assessed for urinary nature. The possibility of urethral communication is therefore presumed, based on previous reports and the concurrent bacteriuria and IUM in all cases, with the same etiologic agent confirmed in at least two dogs, but remains unverified.

While CT and Magnetic Resonance Imaging are preferred for diagnosing Müllerian abnormalities in humans, Magnetic Resonance Imaging is less practical in veterinary medicine, due to cost, limited access, and prolonged anesthetic requirements [21,33,36]. In our cases, AUS provided an accurate presumptive diagnosis, supporting its role as a first-line diagnostic tool given its wide availability and non-invasiveness. CT allowed comprehensive abdominal assessment and improved surgical planning, otherwise limited by the size of the lesions. Despite higher cost and limited accessibility, CT has proven valuable in providing complementary information that enhances diagnostic confidence and guides surgical management [19,21,33]. Given the rarity and potential underdiagnosis of IUM, our combined AUS and CT findings offer novel and valuable insights, while surgery, histopathology, fluid analysis, and culture remain essential for definitive confirmation.

## 5. Conclusions

In conclusion, IUM should be considered in male dogs presenting with abdominal distension, pain, or systemic infection. Both AUS and CT are valuable diagnostic tools, with CT enhancing diagnostic confidence and aiding in surgical planning. In cases with a compatible clinical history, recognition of the diagnostic imaging features described here, including cervix-like mural narrowing or fluid–fluid levels, may help clinicians identify this rare condition. This study also highlights the malignant potential of the UM and the occurrence of diverse gonadal tumors. A comprehensive DSD work-up, including hormonal and genetic testing, was not performed and remains desirable in future studies on UM.

## Figures and Tables

**Table 1:** Signalment, clinical signs, physical examination, and blood laboratory findings.

Case	Signalment	Clinical Signs and Physical Examination	Blood Laboratory Findings
1	10-year-old, intact Miniature Schnauzer 9.4 kg	Acute vomiting, diarrhea, tenesmus, anorexia Polyuria and polydipsia Abdominal pain and distension Hyperthermia (39.1 °C)	Leukocytosis, band neutrophils Anemia Thrombocytopenia Increased alkaline phosphatase
2	14-year-old, intact Biewer Yorkshire Terrier 5.5 kg	Acute vomiting, diarrhea, and anorexia Abdominal pain and distension	Neutropenia, band neutrophils, monocytosis Increased alkaline phosphatase
3	9-year-old, unilaterally (left) neutered Miniature Schnauzer 9.1 kg	Acute anorexia Polyuria and polydipsia Hematuria and pyuria Abdominal pain and distension Hyperthermia (40.3 °C) Gynecomastia	Neutropenia, band neutrophils Anemia Thrombocytopenia Increased globulins and proteins

## Data Availability

The data presented in this study are not publicly available due to privacy protection.

## Abbreviations

The following abbreviations are used in this manuscript:

AUS	Abdominal ultrasonography
CT	Computed tomography
DSD	Disorder of sexual development
IUM	Infected uterus masculinus
PMDS	Persistent Müllerian duct syndrome
UM	Uterus masculinus
UTI	Urinary tract infection
